# The “Dental Digest” Hysterical

**Published:** 1905-04

**Authors:** 


### THE "DENTAL DIGEST" HYSTERICAL.

In the February issue of the *Dental Digest*, of Chicago, is an editorial entitled "Pretence *versus* Practice," in which the editor abuses the International Dental Journal and its editor in language unworthy any respectable journal. The reasons for this sudden attack, from a commercial dental journal, are not as yet made clear, but the article is so intrinsically bad that it carries its own condemnation with it. For this reason the writer declines to notice the personal portion of this hysterical outburst.

The reflections made upon the honesty and ethical character of the International Dental Journal require a definite and positive answer. This journal claims many good friends in the dental profession, who have in the past been equally interested in the so-called Dental Protective Association, and have stood earnestly and persistently by the proprietor of the *Dental Digest* in his strenuous efforts to maintain that Association financially and professionally. That the judgment of these friends may not be warped, it is deemed advisable to notice certain parts of this article. The fact is fully appreciated, however, that a dignified silence is always the best answer to charges that have lost the elements of self-respect.

1. He, the editor of the *Dental Digest*, charges that the International Dental Journal has made "unwarranted personal attacks" upon his journal and those connected with it. This charge has *no foundation in fact*. This the readers of the International can verify by consulting the past volumes. The *Digest* has never been a subject for criticism directly or indirectly.

2. The charge is made that the editor of the International Dental Journal published a paper written by Dr. Anema, and which was considered to be "the property" of the Pennsylvania State Dental Society. The answer to this must be found in the fact that the editor of the journal named received the paper from the author with the request that it be published. This was done, the author presumably feeling that he owned the paper. The present writer has not now, nor ever had, any information as to the cash compensation paid Anema for said paper when it, with other matter, passed over to the *Digest* for a consideration.

3. The International Dental Journal is charged with having published, "without a line of credit," an article entitled "The Pathogeny of Osteomalacia, or Senile Atrophy." It is true that said article found a place upon its pages, having been published in the January issue, 1905. This article was taken from a medical journal sent by direction of the author, and with his consent it was transferred to the department of "Reviews of Dental Literature." The *Dental Digest* was not known in the transaction from beginning to end. The cuts for the illustrations were furnished by the author. The paper was regarded as a very valuable one and eminently worthy of extended circulation.

4. The charge is further made that the editor of the International Dental Journal has for months sold "*his* space to a professional ad. writer." Those interested can refer to this advertisement in recent numbers. It will be found in a very modest corner of its advertising pages. It is simply an announcement that a writer offers his services in the preparation of advertisements. The professional writer of ads. has become an apparent necessity in the business world of the day, and, while this may be abused, it is neither unethical nor criminal.

The editor of the International Dental Journal neither owns that periodical, nor has he, *directly or indirectly*, anything to do with the business management, and consequently has no authority in the acceptance or declination of advertisements.

With an apology to our readers for taking up space in allusion to this scurrilous article, we pass from the subject to more congenial matters.

